# The requirement of mitochondrial RNA polymerase for non-small cell lung cancer cell growth

**DOI:** 10.1038/s41419-021-04039-2

**Published:** 2021-07-29

**Authors:** Tong Zhou, Yong-Hua Sang, Shang Cai, Chun Xu, Min-hua Shi

**Affiliations:** 1 Department of Respiratory and Critical Care Medicine, The Second Affiliated Hospital of Soochow University, Suzhou, China; 2 Department of Thoracic Surgery, The Second affiliated Hospital of Soochow University, Suzhou, China; 3 Department of Radiotherapy and Oncology, The Second Affiliated Hospital of Soochow University, Suzhou, China; 4 Department of Cardio-Thoracic Surgery, The First Affiliated Hospital of Soochow University, Suzhou, China

**Keywords:** Non-small-cell lung cancer, Oncogenes

## Abstract

POLRMT (RNA polymerase mitochondrial) is responsible for the transcription of mitochondrial genome encoding key components of oxidative phosphorylation. This process is important for cancer cell growth. The current study tested expression and potential functions of POLRMT in non-small cell lung cancer (NSCLC). TCGA cohorts and the results from the local lung cancer tissues showed that POLRMT is overexpressed in human lung cancer tissues. In both primary human NSCLC cells and A549 cells, POLRMT silencing (by targeted lentiviral shRNAs) or knockout (through CRSIPR/Cas9 gene editing method) potently inhibited cell viability, proliferation, migration, and invasion, and induced apoptosis activation. On the contrast, ectopic overexpression of POLRMT using a lentiviral construct accelerated cell proliferation and migration in NSCLC cells. The mtDNA contents, mRNA levels of mitochondrial transcripts, and subunits of respiratory chain complexes, as well as S6 phosphorylation, were decreased in POLRMT-silenced or -knockout NSCLC cells, but increased after ectopic POLRMT overexpression. In vivo, intratumoral injection of POLRMT shRNA adeno-associated virus (AAV) potently inhibited NSCLC xenograft growth in severe combined immune deficiency mice. The mtDNA contents, mRNA levels of mitochondria respiratory chain complex subunits, and S6 phosphorylation were decreased in POLRMT shRNA AAV-injected NSCLC xenograft tissues. These results show that POLRMT is a novel and important oncogene required for NSCLC cell growth in vitro and in vivo.

## Introduction

Lung cancer is one common malignancy around the world [1, 2]. It is an extremely aggressive disease with a 5-year overall survival less than 15% [1, 2]. In 2019 over 228,000 new cases of lung cancer and 142,000 mortality cases were reported in the United States alone. It therefore is the leading cause of cancer-associated mortality [1, 2]. Based on pathological classification, lung cancer is roughly divided into small cell lung cancer (SCLC) and non-small cell lung cancer (NSCLC) [3, 4]. Of which, NSCLC accounts over 85% of all cases [3, 4].

A large proportion of NSCLC patients are diagnosed at advanced stages, or relapse after curative-intent surgery [3, 4]. Current standard treatments, including surgery, chemotherapy, immunotherapy, and the molecularly targeted therapies (including the small molecule tyrosine kinase inhibitors) have failed to significantly improve the overall survival for advanced and recurrent NSCLC patients [5–7]. Studies have shown that numerous aberrantly expressed genes and signaling pathways are critically involved in the pathogenesis, tumorigenesis, progression, and therapy-resistance of NSCLC [5–9]. To further explore the underlying mechanisms and novel therapeutic targets for NSCLC is therefore extremely important [5–7].

Mitochondria are responsible for providing cellular energy through the well-known oxidative phosphorylation (OXPHOS) system whereby energy is converted to adenosine 5′-triphosphate [10–14]. This will require transcription and expressions of mitochondrial DNA (mtDNA) [12, 15–17]. Indeed, mtDNA encodes at least 13 essential subunits of the OXPHOS system, 2 mitochondrial ribosomal RNAs, and 22 transfer RNAs [17, 18]. POLRMT (RNA polymerase mitochondrial) along with two other key components, mitochondrial transcription factors A (TFAM) and B2 (TFB2M), is absolutely required for initiating the promoter-specific transcription of mtDNA [19, 20]. POLRMT is also a core component of the mitochondrial transcription machinery [19, 20]. Furthermore, it is involved in the synthesis of RNA primers that are required for the initiation of mtDNA replication [21, 22].

Increased mtDNA transcription for the biogenesis of the OXPHOS system is vital for the growth of rapidly dividing cancer cells [12, 23, 24]. Conversely, targeting OXPHOS in mitochondria should be an important strategy to inhibit cancer cell growth [10, 12, 15, 25]. Studies have shown that POLRMT could be an important metabolic oncogene, promoting OXPHOS progression and cancer cell growth [15, 25–27]. Its expression and potential functions in NSCLC are examined in the present study.

## Materials and methods

### Chemicals, reagents, and antibodies

Puromycin and polybrene were purchased from Sigma (St. Louis, Mo). Cell counting kit-8 (CCK-8) was purchased from Dojindo (Kumamoto, Japan). 5-Ethynyl-2’-deoxyuridine (EdU), 4’,6-diamidino-2-phenylindole (DAPI), terminal deoxynucleotidyl transferase dUTP nick end labeling (TUNEL), and 5,5’,6,6’-tetrachloro-1,1’,3,3’-tetraethyl-imidacarbocyanine (JC-1) dyes were obtained from Thermo Fisher Invitrogen (Shanghai, China). The anti-POLRMT antibody was purchased from Abcam (ab228576, Shanghai, China). Antibodies for p-S6 Ribosomal Protein (Ser235/236, #2211), S6 (2217), cleaved-caspase antibody sampler Kit (#9929), GAPDH (5174), and β-Tubulin (#2146) were purchased from Cell Signaling Technologies (Beverly, MA). From Hyclone (Suzhou, China) fetal bovine serum (FBS), antibiotics, and other cell culture reagents were obtained.

### Human tissues

The lung cancer tissues and matched surrounding lung epithelial tissues from a total of ten written-informed consent primary NSCLC patients (all male, 48–74 years old, stage III–IV) were enrolled. Patients received no prior treatment before surgery. Fresh tissues were obtained at the time of surgery, stored in liquid nitrogen before further biomedical analyses. Each patient provided the written-informed consent. The protocols of this study were approved by the Ethics Committee of Soochow University, in according with the Declaration of Helsinki.

### Cell culture

A549 cell line was from Dr Li at Wenzhou Medical University [28, 29] and cells was cultivated in RPMI-1640 medium containing 8% FBS in a humidified atmosphere containing 5% CO_2_. Using the described protocols [30, 31], the primary human NSCLC cells were derived from three different patients, pNSCLC1, pNSCLC2, and pNSCLC3. Briefly, the fresh NSCLC tissues were minced and digested, and blood vessel cells, immune cells, and fibroblasts were abandoned. The primary cancer cells were re-suspended and maintained in high glucose DMEM/F-12 growth medium with 12% FBS, along with epidermal growth factor (EGF, 1.5 ng/mL) and insulin (5 ng/mL). Generating the primary human lung epithelial cells was through the same protocol except the fresh normal lung epithelial tissues were utilized. Mycoplasma-microbial contamination examination, STR profiling, population doubling time, and morphology were checked to confirm the genotypes. The written-informed consent was obtained from each enrolled patient. The protocols of this study were approved by the Ethics Committee of the Soochow University, in according with the Declaration of Helsinki.

### Quantitative real-time PCR (qRT-PCR)

Trizol reagents (Life Technologies, Gaithersburg, MD) were utilized to extract total RNAs from cultured cells and tissues. Complementary DNA (cDNA) was synthesized using the TaqMan Reverse Transcription Kit (Applied Biosystems, Beijing, China). Applied Biosystems Prism 7900 Fast Real-Time PCR system was employed for the qRT-PCR assays using a SYBR-Green kit (Applied Biosystems). The relative mRNA expression was examined using a standard 2^−ΔΔCt^ method after normalizing to *glyceraldehyde-3-phosphate dehydrogenase* (*GAPDH*). The mRNA primers for *POLRMT* and the subunits of mitochondrial respiratory chain complexes, including *NADH*:*ubiquinone oxidoreductase subunit B8* (*NDUFB8*), *ubiquinol-cytochrome C reductase core protein 2* (*UQCRC2*), and *mitochondrially encoded cytochrome C oxidase I* (*COXI*), as well as for mitochondrial RNAs (mtRNAs), *mitochondrially encoded NADH:ubiquinone oxidoreductase core subunit 1* (*ND1*) and *mitochondrially encoded cytochrome B* (*CYB*), are listed in Table 1.

**Table 1:** Sequences utilized in this study.

Gene name	qRT-PCR primer forward (5’-3’)	qRT-PCR primer reverse (5’-3’)
*POLRMT*	GGACTCCAAGGTCAAGCAAATAGGAG	AGGTCGAAGGCCCCTGGCTTG
*ND1*	CCACCTCTAGCCTAGCCGTTTA	GGGTCATGATGGCAGGAGTAAT
*UQCRC2*	AATTTCGTCGTTGGGAAGTAGC	ATGAGTCTGCGGATTCTGAAAG
*NDUFB8*	TACAACAGGAACCGTGTGGA	CTGGTTCTTTGGAGGGATCA
*CYB*	ATCACTCGAGACGTAAATTATGGCT	TGAACTAGGTCTGTCCCAATGTATG
*COX1*	TCTCAGGCTACACCCTAGACCA	ATCGGGGTAGTCCGAGTAACGT
*GAPDH*	GGAGCGAGATCCCTCCAAAAT	GGCTGTTGTCATACTTCTCATGG
**sgRNA**	**Target DNA sequence**	**PAM sequence**
POLRMT sgRNA	TCTCCAGTATCTTTGCCCAG	CGG

### Western blotting

Cells or tissues were incubated with the RIPA lysis buffer (Beyotime Biotechnology, Wuxi, China) and the quantified protein lysates were separated on 10–12.5% PAGE mini-gels (Bio-Rad Laboratories). The semi-dry transfer system (Trans-Blot SD, Bio-Rad) was utilized for transfer lysate proteins to PVDF membranes (Millipore, Shanghai, China). After blocking (in 10% milk in PBST solution), the blots were incubated with the applied primary antibodies and corresponding secondary antibodies. A SuperSignal ECL system (Thermo Fisher, Shanghai, China) was applied to detect the targeted protein band based on the molecular weights.

### POLRMT shRNA

Lentivirus expressing targeted shRNA against POLRMT was generated using the GV369 construct from Shanghai Genechem Co (Shanghai, China). The non-overlapping shRNA sequences targeting *PLORMT* were individually inserted into the GV369 construct. As the negative control, the scramble non-sense control shRNA (“scr”) was inserted to the construct. Cloning was confirmed by DNA sequencing. To generate lentivirus, the shRNA construct was transfected to HEK-293T cells along with the lentivirus package constructs, pMD2.G and psPAX2 (Genechem, Shanghai, China), and allowed to incubate for 36 h. Cells were then switch into complete medium and cultured for additional 24 h. Medium was harvested and concentrated. For infection, virus was added to NSCLC cells in polybrene (5 μg/mL)-containing medium for 24 h. Afterwards, medium was removed and replaced with complete medium containing 5 μg/mL puromycin for four passages. Expressions of *POLRMT* mRNA and protein in stable cells were verified by qRT-PCR and western blotting assays. For in vivo studies, PLORMT shRNA sequence was packaged into the adeno-associated virus (AAV) construct (AAV9) obtained from Genechem (Shanghai, China). The construct was transfected to HEK-293 cells, generating PLORMT shRNA AAV. The virus was then filtered, enriched, and injected to NSCLC xenografts.

### POLRMT knockout (KO)

A CRISPR/Cas9-POLRMT-KO construct encoding the sgRNA specifically targeting *POLRMT* (listed in Table 1) was produced and verified by Genechem (Shanghai, China). The construct was transfected to pNSCLC1 cells. Afterwards, cells distributed into 96-well plates and subjected to screening of *POLRMT* KO. Single stable POLRMT KO pNSCLC1 cells, or ko-POLRMT cells, were then established. Control cells were transduced with the CRISPR/Cas9 control construct (“Cas9-C”).

### PLORMT overexpression

The full-length PLORMT cDNA was inserted into the lentiviral GV369 construct. The construct was transfected to HEK-293T cells along with the lentivirus package constructs, pMD2.G and psPAX2 (Genechem, Shanghai, China). The generated lentivirus was added to primary NSCLC cells that were cultured in polybrene (5 μg/mL)-containing medium. Puromycin was added to select stable cells. Overexpression of POLRMT was verified by qRT-PCR and western blotting assays.

### Viability assay

NSCLC cells with applied genetic modifications were seeded in 96-well plates at a density of 3.5 × 10^3^ cells per well. Following incubation for 96 h, 15 µL of CCK-8 reagent was added into each well for 2 h before analyzing the absorbance at 450 nm. CCK-8 optical density (OD) was recorded.

### Colony formation

NSCLC cells with applied genetic modifications were initially seeded into 10-cm tissue-culturing dishes at 1 × 10^4^ cells per well and were maintained under complete medium (with 8% FBS). After 10 days, cell colonies were fixed, stained, and counted manually.

### EdU staining

NSCLC cells with applied genetic modifications were seeded in 24-well plates at a density of 1.5 × 10^4^ cells per well and were cultured for 96 h. Afterwards, cells were fixed and permeabilized with 1% formaldehyde and 0.2 % Triton X-100, respectively. EdU and DAPI fluorescence dyes were added, and cell nuclei were visualized under the TE200-U fluorescence microscope (NIKON, Tokyo, Japan). EdU-positive nuclei ratio (% vs. DAPI) was recorded.

### Transwell assays

Briefly, 24-well “Transwell” chambers (12-μm pore, Corning, New York, NY) were utilized for cell migration and invasion assays. NSCLC cells (1.2 × 10^4^ cells per chamber, in 200 µL of DMEM plus 0.5% FBS) with applied genetic modifications were seeded into the upper chambers. The lower chambers were filled with complete medium. After 24 h of incubation, the non-migrated cells at the upper surface were gently removed, while the migrated cells on the lower surface were fixed with 95% alcohol and stained with 1% crystal violet. Images were captured, and the cells were counted. Protocols for the invasion assays were the same except the upper chambers were coated with Matrigel (BD Biosciences, Shanghai, China).

### Caspase-3 activity assay

The caspase-3 activity assay kit was acquired from Solarbio (Beijing, China). In brief, protein extracts (40 μg per treatment) were incubated with caspase reaction buffer and the caspase-3 substrate at 37 °C for 3 h. The caspase-3 activity was examined by detecting the absorbance at 405 nm.

### DNA breaks

NSCLC cells with applied genetic modifications were seeded in 96-well plates at a density of 3.5 × 10^3^ cells per well. Following incubation for 72 h, a single-strand DNA (ssDNA) ELISA kit (Merck Millipore, Shanghai, China) was employed to test ssDNA contents, and ELISA OD at 450 nm in each well was recorded.

### TUNEL assay of cell apoptosis

NSCLC cells with applied genetic modifications were seeded in 24-well plates at a density of 2.0 × 10^4^ cells per well and were cultured for 96 h. Cells were then fixed and permeabilized, and TUNEL and DAPI dyes were added. Cell nuclei were then visualized by TE200-U fluorescence microscope. TUNEL-positive nuclei ratio (% vs. DAPI) was recorded.

### Measurement of mitochondrial membrane potential (MMP)

The MMP was evaluated using a JC-1 staining kit (Beyotime, Shanghai, China). JC-1 could form green monomers in cells with MMP reduction and mitochondrial depolarization. Briefly, NSCLC cells with applied genetic modifications were plated into 6-well plates at a density of 6 × 10^4^ cells per well. Cells were cultured for 72 h and incubated with 2 μM of JC-1 at 37 °C for 1 h. JC-1 green monomer intensity was recorded and representative JC-1 fluorescence images were taken.

### Determination of mtDNA levels by quantitative PCR (qPCR)

Total DNA was extracted and purified using a Qiagen kit (Qiagen, Shanghai, China). The purity and quantity of DNA were examined using the NanoDrop 2000 (Thermo Scientific). For each treatment 5 ng/μL DNA was analyzed by qPCR using the Taqman 2× Universal PCR mastermix (Applied Biosystems) and commercially available Taqman assay probes for *COX1* mtDNA (18S). Its level was normalized to control.

### NSCLC xenograft study

Severe combined immune deficiency (SCID) mice, half male and hale female, 10- to 11-week of age, 18.5–9.5 g of weights, were obtained from the experimental animal center of Soochow University (Suzhou, China). Mice were housed in a 12 h light/dark cycle in controlled environmental conditions (22.3 ± 2 °C, 44–58% relative humidity) and fed with a normal chow diet and water ad libitum. For xenograft assay, mice were subcutaneously injected with pNSCLC1 cells (5 × 10^6^ cells in 100 μL of PBS and Matrigel per mouse). Within 20–22 days NSCLC xenograft-bearing SCID mice were established and the volume of each xenograft was close to 100 mm^3^. The mice were then randomized into two groups: ten mice were intratumorally injected with POLRMT shRNA AAV and the other ten mice received control shRNA AAV. Mice were injected AAV daily for 7 days. Tumor dimensions were measured by calliper every 7 days and volume was estimated as per: *V* = length × width × height × 0.5236. All animal studies were carried out according to the regulations of the IACUC and with the approval of the Institute Animal Ethics Review Board of Soochow University.

### Statistical analysis

The investigators were always blinded to the group allocation during all experiments. Numeric data (normal distribution) were expressed as mean ± standard deviation (SD). Statistical differences between multiple groups were analyzed by one-way analysis of variance followed by Dunnett’s test after these data were confirmed to have a normal distribution (SPSS 23.0, Chicago, CA). Unpaired Student’s *t*-test was employed to compare two groups (Excel 2010). A *P* value of <0.05 was considered to indicate a statistically significant result.

## Results

### POLRMT overexpression in human lung cancer tissues and cells

**Fig. 1:**
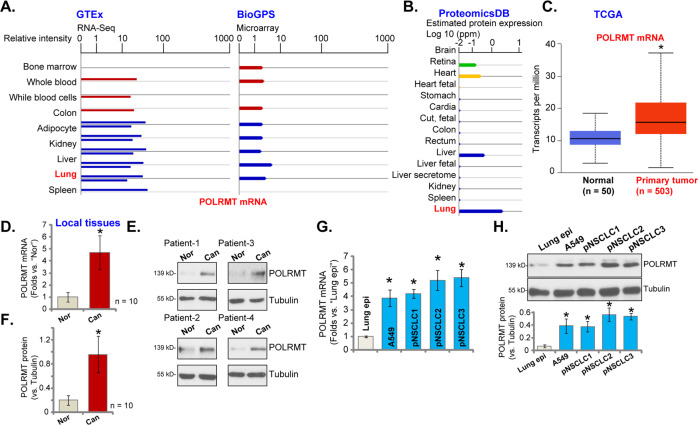
POLRMT overexpression in human lung cancer tissues and cells. Results from the GTEx mRNA expression database and BioGPS microarray expression database demonstrated the relative *POLRMT* mRNA in lung and other human tissues (**A**). ProteomicsDB protein expression database demonstrated the relative POLRMT protein expression in lung and other human tissues (**B**). The elative *POLRMT* mRNA expression in lung cancer tissues (“Primary cancer”, *n* = 503) and normal lung tissues (“Normal”, *n* = 50) in TCGA cohorts (**C**). *POLRMT* mRNA and protein expressions in local human NSCLC cancer tissues (“Can”) and matched surrounding normal lung tissues (“Nor”) from ten primary NSCLC patients were shown, and results were quantified (**D**–**F**). *POLRMT* mRNA and protein expressions in listed NSCLC cells and primary lung epithelial cells (“Lung epi”) were shown, and results were quantified (**G**, **H**). Data were presented as mean ± standard deviation (SD). *P < 0.05 vs. “Normal” tissues (**C**), “Nor” tissues (**D**, **F**), and “Lung epi” cells (**G**, **H**).

We first searched the Genotype-Tissue Expression (GTEx) mRNA expression database and BioGPS microarray expression database and found that *POLRMT* mRNA expression is detected in normal lung tissues (Fig. 1A). In addition, results from the protein-centric in-memory database ProteomicsDB [32] show that POLRMT protein is expressed in human lung tissues (Fig. 1B). The Cancer Genome Atlas (TCGA) cohorts showed a significantly high expression of *POLRMT* mRNA in lung cancer tissues (“Primary tumor”, *n* = 503) (Fig. 1C). A relatively low expression in normal lung tissues (“Normal”, *n* = 50) was detected (Fig. 1C).

To further confirm the bioinformatics results, qRT-PCR assays were performed to examine the expression levels of *POLRMT* mRNA in the local human lung cancer tissues (“Can”) and matched surrounding normal lung tissues (“Nor”) from ten primary NSCLC patients. As shown, *POLRMT* mRNA expression in cancer tissues was significantly higher than that in the normal tissues (Fig. 1D). Testing POLRMT protein expression, by western blotting assays, demonstrated that POLRMT is upregulated in four representative NSCLC patients (“Patient-1 to Patient-4”, Fig. 1E). When combining all ten sets of human tissues, we found that a significant upregulation of POLRMT protein was detected (*P* < 0.05 vs. “Nor” tissues) (Fig. 1F).

We also tested expression of POLRMT in NSCLC cells. In primary human NSCLC cells derived from three different patients (pNSCLC1, pNSCLC2, and pNSCLC3) and the established A549 cells, *POLRMT* mRNA (Fig. 1G) and protein (Fig. 1H) expressions were significantly higher than those in the primary lung epithelial cells (“Lung epi”) (Fig. 1G, H). Together, these results show that POLRMT is overexpressed in human lung cancer tissues and cells.

### POLRMT shRNA suppresses NSCLC cell growth, proliferation, and migration

**Fig. 2:**
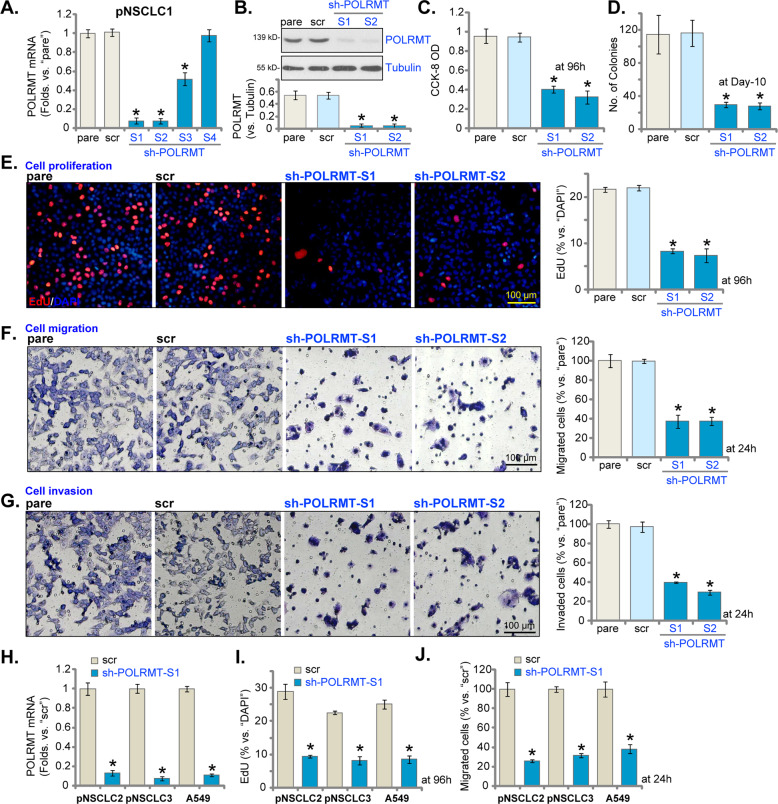
POLRMT shRNA suppresses NSCLC cell growth, proliferation, and migration. The primary human NSCLC cells (pNSCLC1, pNSCLC2, and pNSCLC3) or established A549 cells, expressing the applied POLRMT shRNA (“sh-POLRMT-S1/S2/S3/S4”) or scramble non-sense control shRNA (“scr”), were established, and expressions of *POLRMT* mRNA and listed proteins were tested by qRT-PCR and western blotting assays (**A**, **B**, **H**). Cells were further cultured for applied time periods, and cell viability, proliferation, migration, and invasion were tested by the listed assays mentioned in the text, and results were quantified and normalized (**C**–**G**, **I**, **J**). “pare” stands for parental control cells. Data were presented as mean ± standard deviation (SD, *n* = 5). *P < 0.05 vs. “scr” cells. The experiments were repeated five times with similar results obtained. Scale bar = 100 μm (**E**–**G**).

To understand the potential function of POLRMT in NSCLC cells, shRNA method was employed to silence POLRMT. A set of four different lentiviral shRNAs, targeting non-overlapping sequences of *POLRMT* (sh-POLRMT-S1/S2/S3/S4), were individually transduced into the primary human NSCLC cells (pNSCLC1, see Materials and methods). Stable cells with POLRMT shRNA were established by puromycin selection. The qRT-PCR results, Fig. 2A, demonstrated that three (sh-POLRMT-S1/S2/S3) out of the four tested shRNAs induced significant *POLRMT* mRNA silencing in pNSCLC1 cells. Of which sh-POLRMT-S1 and sh-POLRMT-S2 presented with highest efficiency in silencing *POLRMT* (Fig. 2A). Western blotting assay results, Fig. 2B, demonstrated that POLRMT protein levels were dramatically downregulated in pNSCLC1 cells expressing sh-POLRMT-S1 and sh-POLRMT-S2. To study the functional consequence of POLRMT silencing in NSCLC cells, CCK-8 viability assay was applied. As shown, shRNA-induced silencing of POLRMT significantly decreased CCK-8 viability OD in pNSCLC1 cells (Fig. 2C). In addition, the number of viable pNSCLC1 cell colonies was dramatically decreased in POLRMT-silenced pNSCLC1 cells (Fig. 2D).

EdU incorporation assays were performed to test cell proliferation. In pNSCLC1 cells, after POLRMT silencing the EdU-positive nuclei ratio was significantly decreased (Fig. 2E). Moreover, shRNA-induced knockdown of POLRMT largely inhibited pNSCLC1 cell migration and invasion, which were tested by “Transwell” (Fig. 2F) and “Matrigel Transwell” (Fig. 2G) assays, respectively. In pNSCLC1 cells, transfection of the scramble non-sense control shRNA (“scr”) did not alter *POLRMT* mRNA and protein expressions (Fig. 2A, B) as well as pNSCLC1 cell functions (Fig. 2C–G).

Whether POLRMT silencing exerted similar activity in other NSCLC cells was studied next. The primary human NSCLC cells derived from two other primary patients, pNSCLC2 and pNSCLC3, as well as established A549 cells, were stably transfected with sh-POLRMT-S1 lentivirus. The latter resulted in profound *POLRMT* mRNA downregulation (Fig. 2H). Confirming proliferation inhibition consequence, we showed that sh-POLRMT-S1 dramatically decreased the EdU-positive nuclei ratio in NSCLC cells (Fig. 2I). “Transwell” assay results further demonstrated that POLRMT silencing inhibited migration of the primary and established NSCLC cells (results quantified in Fig. 2J). These results showed that NSCLC cell viability, proliferation, migration, and invasion were largely inhibited with POLRMT silencing.

### POLRMT shRNA provokes apoptosis activation in NSCLC cells

**Fig. 3:**
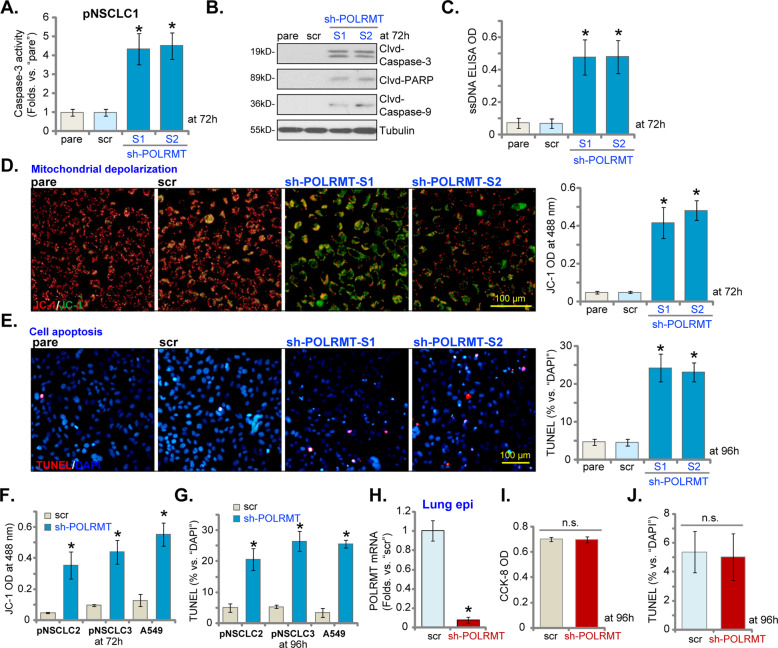
POLRMT shRNA provokes apoptosis activation in NSCLC cells. The primary human NSCLC cells (pNSCLC1, pNSCLC2, and pNSCLC3) or established A549 cells, expressing the applied POLRMT shRNA (“sh-POLRMT-S1/S2/S3/S4”) or scramble non-sense control shRNA (“scr”), were established and cultured for applied time periods, the relative caspase-3 activity (**A**), caspase-PARP cleavages (**B**), ssDNA contents (ELISA OD, **C**), and mitochondrial depolarization (JC-1 assays, **D**, **F**) were tested, and results were quantified and normalized. Cell apoptosis was tested by nuclear TUNEL staining assays (**E**, **G**), with results quantified. The primary human lung epithelial cells (“Lung epi”), expressing the sh-POLRMT-S1 (“sh-POLRMT”) or scramble non-sense control shRNA (“scr”), were established. *POLRMT* mRNA was tested by qRT-PCR assays (**H**). Cells were further cultured for applied time periods, and cell viability and apoptosis were tested by CCK-8 (**I**) and TUNEL staining (**J**) assays, respectively, and results were quantified. “pare” stands for parental control cells. Data were presented as mean ± standard deviation (SD, *n* = 5). *P < 0.05 vs. “scr” cells. n.s. stands for non-statistical difference (**I**, **J**).The experiments were repeated five times with similar results obtained. Scale bar = 100 μm (**D**, **E**).

Next, experiments were performed to test whether POLRMT silencing could provoke apoptosis activation in NSCLC cells. As compared to scr pNSCLC1 cells (see Fig. 2), the relative caspase-3 activity was significantly increased in pNSCLC1 cells expressing POLRMT shRNAs (Fig. 3A). Furthermore, increased cleavages of caspase-3, caspase-9 and poly (ADP-ribose) polymerase (PARP) were detected in pNSCLC1 cells with sh-POLRMT-S1/S2 (Fig. 3B). Indicating DNA damage, we showed that the single-strand DNA contents (ELISA OD) were significantly increased in POLRMT shRNA pNSCLC1 cells (Fig. 3C). In addition, POLRMT shRNA-induced mitochondrial depolarization in pNSCLC1 cells, the latter was evidenced by JC-1 green monomers accumulation [33] (Fig. 3D). These results implied the activation of mitochondrial apoptosis cascade [34–37] in POLRMT-silenced pNSCLC1 cells.

We showed that POLRMT shRNA-induced significant apoptosis activation in pNSCLC1 cells (Fig. 3E). Apoptosis activation was evidenced by increased TUNEL-positive nuclei ratio (Fig. 3E) in POLRMT-silenced pNSCLC1 cells. The lentiviral scramble non-sense control shRNA (“scr”) failed to induce apoptosis activation in pNSCLC1 cells (Fig. 3A–E). Significantly, in A549 cells and other primary NSCLC cells (pNSCLC2 and pNSCLC3, see Fig. 2), sh-POLRMT-S1-induced silencing of POLRMT led to mitochondrial depolarization (JC-1 green monomer intensity increase, Fig. 3F) and apoptosis activation (TUNEL-positive nuclei ratio increase, Fig. 3G). These results showed that POLRMT silencing induced significant apoptosis activation in primary and established NSCLC cells.

In the primary human lung epithelial cells (“Lung epi”, see Fig. 1) stable transfection of sh-POLRMT-S1 lentivirus led to similar *POLRMT* mRNA downregulation (Fig. 3H). However, it failed to induce significant viability reduction (CCK-8 OK) (Fig. 3I) and apoptosis activation (TUNEL staining assay, Fig. 3J) in epithelial cells. These results implied a cancer cell-specific effect by the POLRMT shRNA.

### POLRMT KO potently inhibits NSCLC cell proliferation and induces apoptosis activation

**Fig. 4:**
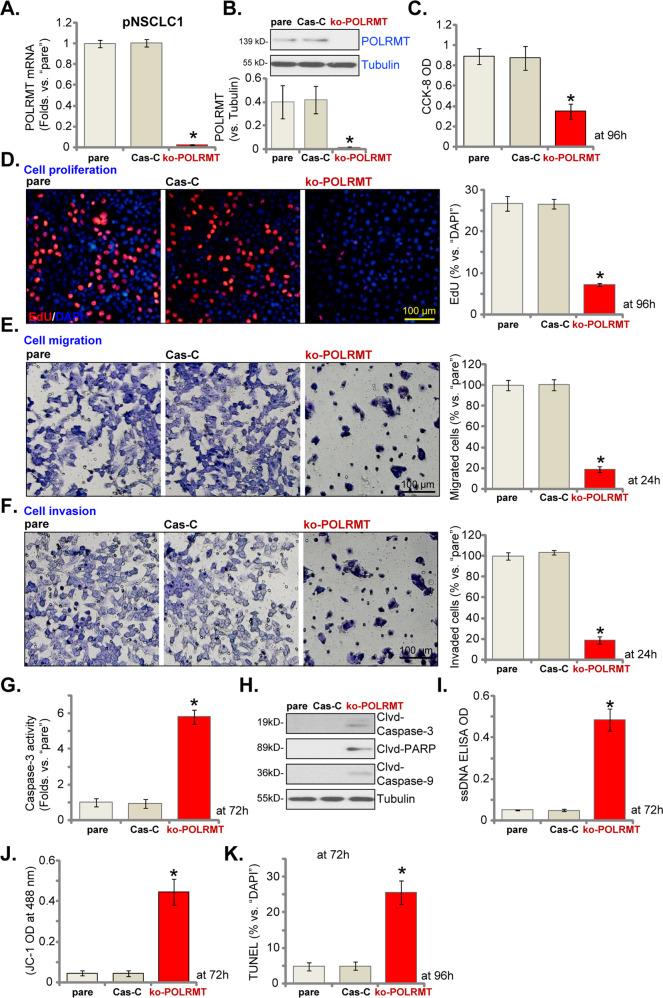
POLRMT knockout potently inhibits NSCLC cell proliferation and induces apoptosis activation. Single stable primary pNSCLC1 cells, expressing the CRISPR/Cas9-POLRMT-KO construct (“ko-POLRMT”) or the CRISPR/Cas9 control construct (“Cas9-C”), were established, and expressions of *POLRMT* mRNA and listed proteins were tested by qRT-PCR (**A**) and western blotting (**B**) assays. Cells were further cultured for applied time periods, and cell viability, proliferation, migration, and invasion were tested by CCK-8 (**C**), EdU incorporation (**D**), “Transwell” (**E**), and “Matrigel Transwell” (**F**) assays, respectively, and results were quantified and normalized. The relative caspase-3 activity (**G**), caspase-PARP cleavages (**H**), and ssDNA contents (ELISA OD, **I**) as well as mitochondrial depolarization (by recording JC-1 green monomers intensity, **J**) and cell apoptosis (by recording TUNEL-positive nuclei ratio, **K**) were tested as well, and results were quantified and normalized. “pare” stands for parental control cells. Data were presented as mean ± standard deviation (SD, *n* = 5). *P < 0.05 vs. “Cas9-C” cells. The experiments were repeated five times with similar results obtained. Scale bar = 100 μm (**D**–**F**).

In order to further confirm the function of POLRMT in NSCLC cells, CRISPR/Cas9 method was utilized to knockout POLRMT. As described, a CRISPR/Cas9-POLRMT-KO construct was transduced to pNSCLC1 cells. Single stable cells with the construct were established via screening: ko-POLRMT cells. As compared to control cells expressing CRISPR/Cas9 construct (Cas9-C), expressions of *POLRMT* mRNA (Fig. 4A) and protein (Fig. 4B) were depleted in ko-POLRMT pNSCLC1 cells. POLRMT KO induced robust viability (CCK-8 OD) reduction (Fig. 4C) and proliferation inhibition (EdU-positive nuclei ratio reduction, Fig. 4D) in pNSCLC1 cells. Furthermore, cell migration and invasion, tested by “Transwell” (Fig. 4E) and “Matrigel Transwell” (Fig. 4F) assays, were also largely inhibited in the ko-POLRMT cells.

In pNSCLC1 cells, CRISPR/Cas9-induced POLRMT KO induced caspase-3 activity increase (Fig. 4G), caspase-3, caspase-9, and PARP cleavages (Fig. 4H) as well as ssDNA accumulation (Fig. 4I). Furthermore, significant mitochondrial depolarization (JC-1 green monomer intensity increase, Fig. 4J) and apoptosis activation (TUNEL-positive nuclei ratio increase, Fig. 4K) were detected in the ko-POLRMT pNSCLC1 cells. Cas9-C construct, as expected, did not alter POLRMT expression and pNSCLC1 cell functions (Fig. 4A–K). Together, these results showed that POLRMT KO inhibited proliferation and induced apoptosis activation in NSCLC cells.

### NSCLC cell proliferation and migration are accelerated after ectopic overexpression of POLRMT

**Fig. 5:**
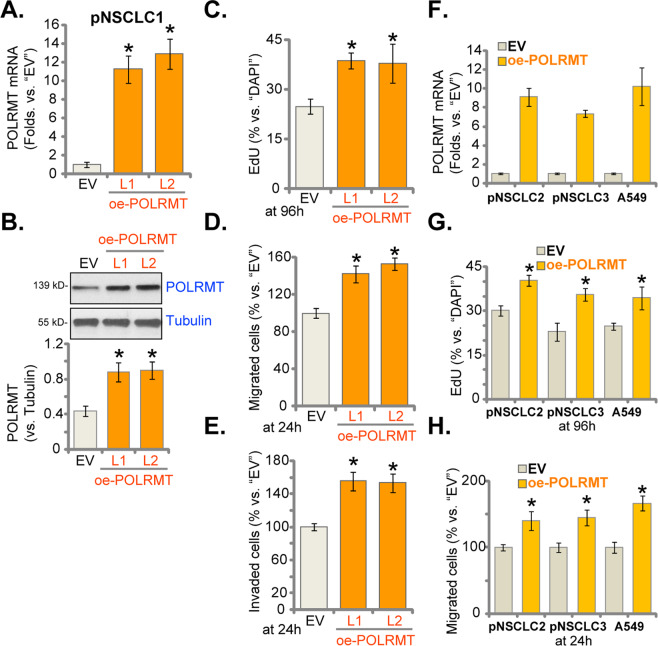
NSCLC cell proliferation and migration are accelerated after ectopic overexpression of POLRMT. The primary human NSCLC cells (pNSCLC1, pNSCLC2, and pNSCLC3) or established A549 cells, expressing the lentiviral construct encoding the full-length POLRMT cDNA (“oe-POLRMT-L1” and “oe-POLRMT-L2,” indicating two different lines) or the empty vector (“EV”) were established. Expressions of *POLRMT* mRNA and listed proteins were tested by qRT-PCR (**A**, **F**) and western blotting (**B**) assays. Cells were further cultured for applied time periods, and cell proliferation, migration, and invasion were tested by EdU incorporation (**C**, **G**), “Transwell” (**D**, **H**), and “Matrigel Transwell” (**E**) assays, respectively, and results were quantified and normalized. Data were presented as mean ± standard deviation (SD, *n* = 5). *P < 0.05 vs. “EV” cells. The experiments were repeated five times with similar results obtained.

Above results have demonstrated that decreasing POLRMT expression, using shRNA or CRISPR/Cas9 strategies, induced proliferation inhibition and apoptosis activation in NSCLC cells. Thus, forced overexpression of POLRMT could possibly exert opposite functions. A lentiviral construct encoding the full-length POLRMT cDNA was transduced to pNSCLC1 cells. Two stable cell lines, “oe-POLRMT-L1” and “oe-POLRMT-L2”, were established via selection by puromycin. As compared to control cells expressing the empty vector (“EV”), *POLRMT* mRNA levels increased over ten folds in the oe-POLRMT pNSCLC1 cells (Fig. 5A). POLRMT protein overexpression was detected as well (Fig. 5B). Testing cell proliferation, by examining EdU-positive nuclei ratio, demonstrated that ectopic overexpression of POLRMT promoted pNSCLC1 cell proliferation (increased EdU-positive nuclei ratio, Fig. 5C). Moreover, quantified results from “Transwell” (Fig. 5D) and “Matrigel Transwell” (Fig. 5E) assays showed that POLRMT overexpression facilitated pNSCLC1 cell migration and invasion.

The lentiviral POLRMT expression construct was also stably transduced to A549 cells and other primary NSCLC cells (pNSCLC2 and pNSCLC3), resulting in robust *POLRMT* mRNA upregulation (“oe-POLRMT” cells) (Fig. 5F). Ectopic overexpression of POLRMT led to augmented cell proliferation (EdU-positive nuclei ratio increase, Fig. 5G) and migration (Fig. 5H) in the NSCLC cells. These results showed that ectopic overexpression of POLRMT could accelerate NSCLC cell proliferation, migration, and invasion, further supporting the oncogenic role of POLRMT in NSCLC cells.

### mtDNA contents, mitochondrial transcripts, subunits of respiratory chain complexes, and S6 phosphorylation are decreased in POLRMT-depleted NSCLC cells

**Fig. 6:**
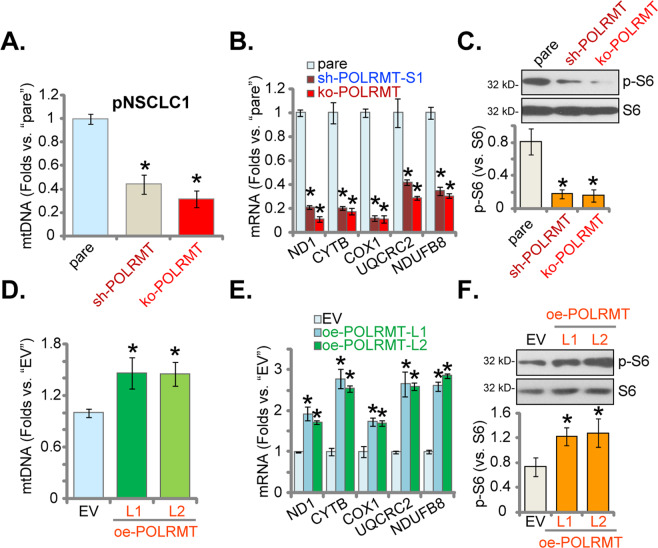
mtDNA contents, mitochondrial transcripts, subunits of respiratory chain complexes, and S6 phosphorylation are decreased in POLRMT-depleted NSCLC cells. Stable pNSCLC1 cells expressing the lentiviral POLRMT shRNA (“sh-POLRMT-S1”), the lentiviral CRISPR/Cas9-POLRMT-KO construct (“ko-POLRMT”), the lentiviral construct encoding the full-length POLRMT cDNA (oe-POLRMT-L1 and oe-POLRMT-L2, two lines), or the empty vector (“EV”) were established and cultured. Levels of mtDNA (**A**, **D**), listed mRNAs (qRT-PCR assays, **B**, **E**), and S6 phosphorylation (**C**, **F**) were tested, and results were quantified and normalized. “pare” stands for parental control cells. Data were presented as mean ± standard deviation (SD, *n* = 5). *P < 0.05 vs. “pare”/“EV” cells. The experiments were repeated five times with similar results obtained.

Studies have shown that POLRMT inhibition resulted in gradual depletion of mtDNA and decreases in the levels of mitochondrial transcripts [15]. We therefore tested mtDNA levels in NSCLC cells with POLRMT silencing/KO. As shown, in the stable pNSCLC1 cells with sh-POLRMT (sh-POLRMT-S1, see Figs 2 and 3) or the CRISPR/Cas9-POLRMT-KO construct (ko-POLRMT, see Fig. 4), levels of mtDNA were significantly decreased when compared to those in the parental control cells (Fig. 6A). The mRNA levels of mitochondrial transcripts (*ND1* and *CYTB*) were decreased as well in sh-POLRMT and ko-POLRMT pNSCLC1 cells (Fig. 6B). In addition, mRNA expression of subunits of respiratory chain complexes, including *NDUFB8*, *UQCRC2*, and *COXI* [15], were reduced after POLRMT silencing or KO (Fig. 6B).

Moreover, S6 phosphorylation was inhibited in POLRMT-depleted pNSCLC1 cells (Fig. 6C). These results showed that POLRMT depletion in pNSCLC1 cells decreased mtDNA contents, mitochondrial transcripts, expressions of subunits of respiratory chain complexes, and S6 phosphorylation. Conversely, when compared to pNSCLC1 cells with empty vector (“EV”), the mtDNA contents (Fig. 6D), mRNA expressions of *ND1*, *CYTB*, *NDUFB8*, *UQCRC2*, and *COXI* (Fig. 6E) as well as S6 phosphorylation (Fig. 6F) were significantly increased in POLRMT-overexpressed pNSCLC1 cells: oe-POLRMT-L1 and oe-POLRMT-L2 (see Fig. 5).

### POLRMT shRNA inhibits pNSCLC1 xenograft growth in SCID mice

**Fig. 7:**
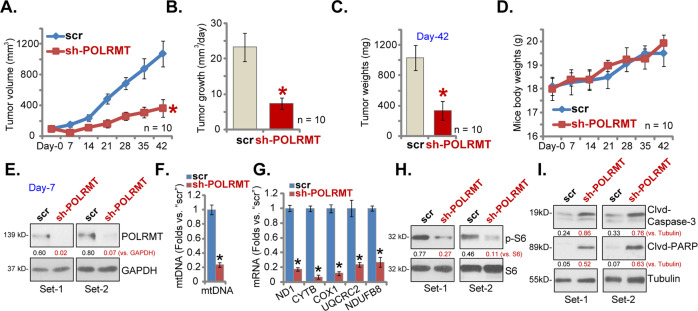
POLRMT shRNA inhibits pNSCLC1 xenograft growth in SCID mice. The pNSCLC1 xenograft-bearing SCID mice were subjected to intratumoral injection of AAV-POLRMT shRNA (“sh-POLRMT”) or AAV-scramble control shRNA (“scr”), daily for 7 days. Tumor volumes (**A**) and mice body weights (**D**) were recorded every 7 days for a total of 42 days (“Day-0 to Day-42”). The estimated daily tumor growth was calculated as described (**B**). All xenografts were isolated at Day-42 and weighted individually (**C**). At Day-7, 6 h after AAV injection, two tumors of each group were isolated. The fresh tumor tissues were subjected to western blotting and qRT-PCR assays to test listed genes (**E**, **G**, **H**, **I**). The mtDNA levels were tested as well (**F**). Expressions of listed proteins were quantified and normalized to the loading controls (**E**, **H**, **I**). Data were presented as mean ± standard deviation (SD). Ten mice were in each group (*n* = 10) (**A**–**D**). *P < 0.05 vs. “scr” group (**A**–**C**, **F**, **G**).

Next we tested the potential effect of POLRMT on the NSCLC cell growth in vivo. NSCLC xenografts were established by subcutaneous injection of pNSCLC1 cells to the flanks of the SCID mice (at 5 × 10^6^ cells per mouse). With 20–22 days the pNSCLC1 xenografts were established and tumor volume was close to 100 mm^3^ in each mouse (labeled as “Day-0”). The xenograft mice were then randomly assigned into two groups (ten mice per group), receiving intratumoral injection of AAV-POLRMT shRNA (“sh-POLRMT”) or AAV-scramble control shRNA (“scr”). AAV injection was performed daily for 7 consecutive days. The weekly tumor growth curve results, Fig. 7A, demonstrated that the growth of sh-POLRMT pNSCLC1 xenografts was significantly slower than scr pNSCLC1 xenografts. The estimated daily tumor growth was calculated by the following formula: [Tumor volume (in mm^3^) at Day-42 subtracting tumor volume (in mm^3^) at Day-0]/42. Results showed that AAV-POLRMT shRNA injection largely inhibited pNSCLC1 xenograft growth in SCID mice (Fig. 7B). All tumors were isolated at Day-42 and weighted individually. We found that sh-POLRMT-injected pNSCLC1 xenografts were significantly lighter than scr xenografts (Fig. 7C). As shown in Fig. 7D, the mice body weights were not significantly different between the two groups.

At Day-7, 6 h after initial AAV injection, two tumors of each group were isolated. Total four tumors were subjected to signaling analyses. Western blotting assays testing fresh tumor tissue lysates, Fig. 7E, confirmed dramatic POLRMT silencing in sh-POLRMT pNSCLC1 xenografts. In addition, levels of mtDNA were decreased in pNSCLC1 xenografts-expressing POLRMT shRNA (Fig. 7F). Furthermore, mitochondrial transcripts (*ND1* and *CYTB*) and mRNA levels of subunits of respiratory chain complexes (*NDUFB8*, *UQCRC2*, and *COXI*) were significantly decreased in sh-POLRMT xenograft tissues (Fig. 7G). S6 phosphorylation was inhibited as well in xenograft tissues with POLRMT shRNA (Fig. 7H). Levels of cleaved-Caspase-3 and cleaved-PARP were, however, increased in AAV-POLRMT shRNA-injected tumor tissues, suggesting apoptosis activation (Fig. 7I). Therefore, the signaling changes in POLRMT-silenced xenografts were consistent with in vitro findings.

## Discussion

Increased mtDNA transcription for the biogenesis of the OXPHOS system in rapidly dividing cancer cells is vital for tumor growth [12, 23, 24]. Targeting mtDNA transcription therefore could produce robust anti-tumor activity [12, 23, 24]. POLRMT is essential for mtDNA transcription and OXPHOS [12, 23, 24]. Recent studies have proposed POLRMT as a novel metabolic oncogene for human cancers [15, 26, 27]. Chaudhary et al. reported that POLRMT is overexpressed in acute myeloid leukemia (AML), which is associated with increased mtDNA copy number and lower patients’ survival [26]. Bralha et al. reported that in AML cells shRNA-induced POLRMT silencing decreased mitochondrial gene expression, OXPHOS, and expressions of subunits of respiratory chain complexes, but increased cell death [27]. Importantly, in AML cells a mitochondrial transcription inhibitor 2-C-methyladenosine (2-CM) largely inhibited mitochondrial gene expression and OXPHOS, causing robust cancer cell death [27]. Bonekamp et al. recently developed a first-in-class and direct POLRMT inhibitor of mitochondrial transcription 1 (IMT1). IMT1 inhibited cancer cell growth in vitro and displayed a robust anti-tumor activity in xenografts of cancer cells [15]. These results implied that in cancer cells POLRMT blockage or silencing impaired mtDNA transcription and OXPHOS, thus inhibiting cancer growth [15, 26, 27].

The results of this study implied that POLRMT could be a novel and important oncogenic gene for NSCLC. TCGA cohorts show that *POLRMT* mRNA expression in lung cancer tissues is significantly higher than that in normal lung tissues. Analyzing local tissue specimens, we found that *POLRMT* mRNA and protein levels in NSCLC tissues are higher than those in the matched surrounding normal lung tissues. Furthermore, POLRMT overexpression was detected in primary (pNSCLC1, pNSCLC2, and pNSCLC3) and established (A549) NSCLC cells. In primary human NSCLC cells and A549 cells, POLRMT silencing or CRISPR/Cas9-mediated KO potently inhibited cell viability, proliferation, migration, and invasion. Mitochondrial depolarization and apoptosis activation were detected in POLRMT-silenced/-KO NSCLC cells. Conversely, cell proliferation, migration, and invasion were accelerated after forced POLRMT overexpression in NSCLC cells. Importantly, intratumoral injection of POLRMT shRNA AAV potently inhibited NSCLC xenograft growth in mice. Therefore, targeting POLRMT could potently inhibit NSCLC cell growth in vitro and in vivo.

In primary NSCLC cells with POLRMT shRNA or KO, levels mtDNA, mitochondrial transcripts (*ND1* and *CYTB* mRNAs) as well as mRNA expressions of subunits of mitochondrial respiratory chain complexes (*COX1*, *UQCRC2*, and *NDUFB8*) were significantly decreased. In the contrast, these parameters were increased in POLRMT-overexpressed NSCLC cells. Furthermore, decreased levels of mtDNA, mitochondrial transcripts, and *COX1*, *UQCRC2*, and *NDUFB8* mRNAs were detected in POLRMT-shRNA AAV-injected NSCLC xenografts. The changes in OXPHOS system detected both in vitro and in vivo could explain the critical importance of POLRMT in rapidly dividing NSCLC cells, and why targeting POLRMT could have substantial anti-tumor effects against NSCLC cell both in vitro and in vivo.

In NSCLC depletion or mutation of multiple genes (*PTEN*, *PI3KCA*, *RTK*, etc.) shall cause dysregulation and overactivation of mammalian target of rapamycin (mTOR), thus promoting tumorigenesis and cancer progression [5, 38]. mTOR overactivation could promote NSCLC cell growth, proliferation, migration, and angiogenesis. It is also associated with apoptosis inhibition and therapy-resistance, and represents as an important therapeutic target of NSCLC [5, 38–41]. Targeted inhibition of mTOR cascade, on the other hand, could produce significant anti-tumor activity in NSCLC cells [29, 38, 41, 42]. Bonekamp et al. found that POLRMT inhibition by small molecular inhibitors could result in robust inhibition of S6 phosphorylation [15], a key indicator of mTORC1 activation. We found that S6 phosphorylation was inhibited in POLRMT-silenced/-KO NSCLC cells and in POLRMT-shRNA AAV-injected NSCLC xenograft tissues, but was increased in NSCLC cells with POLRMT overexpression. Therefore, mTOR inhibition could be another important mechanism for PLORMT depletion-induced anti-tumor activity in NSCLC cells. The underlying signaling mechanisms may warrant further characterizations.

## Conclusion

In recent years significant progresses have been achieved in early screening and diagnosis of NSCLC [5, 7]. NSCLC-targeted therapies are being developed as well. However, for advanced/recurrent NSCLC patients, the overall survival and prognosis are still not satisfied [1, 2]. There is an urgent need to explore novel therapeutic interventions [5, 43]. Our results implied that targeting POLRMT could represent as a novel and fine strategy to inhibit NSCLC cell growth in vitro and in vivo.
